# Supplementary material for: Coordinated regulation of the entry and exit steps of aromatic amino acid biosynthesis supports the dual lignin pathway in grasses
Source: Nat Commun. 2023 Nov 9;14:7242. doi: 10.1038/s41467-023-42587-7 (PMC10636026; doi:10.1038/s41467-023-42587-7)
Supplement: Supplementary file 1 — Supplementary Information [file 41467_2023_42587_MOESM1_ESM.pdf]

SUPPLEMENTAL FIGURES FOR

Coordinated Regulation of the Entry and Exit Steps of Aromatic Amino Acid Biosynthesis Supports the Dual Lignin Pathway in Grasses

El-Azaz et al., 2023

Nature Communications

Supplemental Figure S1. ^{13}C-isotopologue kinetics corresponding to the experiment shown in main Figure 1.

Supplemental Figure S2. ^{13}C-isotopologue kinetics corresponding to the experiment shown in main Figure 2.

Supplemental Figure S3. Transient expression of TyrA-EGFP fusion proteins in Arabidopsis protoplasts.

Supplemental Figure S4. Original Ct values and calibration curves for the RT-qPCR determination of *TyrA* expression in grasses.

Supplemental Figure S5. Substrate and cofactor usage of recombinant grass TyrAs.

Supplemental Figure S6. Michaelis-Menten and tyrosine inhibition plots of grass TyrAs.

Supplemental Figure S7. Inhibition kinetics of BdTyrA1 and BdTyrAnc.

Supplemental Figure S8. Golden-Gate plant expression constructs used in this study.

Supplemental Figure S9. Michaelis-Menten plots of Brachypodium DHSs.

Supplemental Figure S10. Inhibition of Brachypodium DHSs to AAAs downstream metabolites, and UHPLC-MS validation of the results shown in main Figures 4C and 4E.

Supplemental Figure S11. DHS inhibition assay using hydrolyzed arogenate as control.

Supplemental Figure S12. Feedback inhibition of recombinant Sorghum DHSs.

Supplemental Figure S13. Inhibition kinetics of BdDHS2 for tryptophan and arogenate.

Supplemental Figure S14. DHS inhibition assay using combined effectors.

Supplemental Figure S15. Immunoblot quantification of Brachypodium DHSs abundance in *Nicotiana benthamiana*.

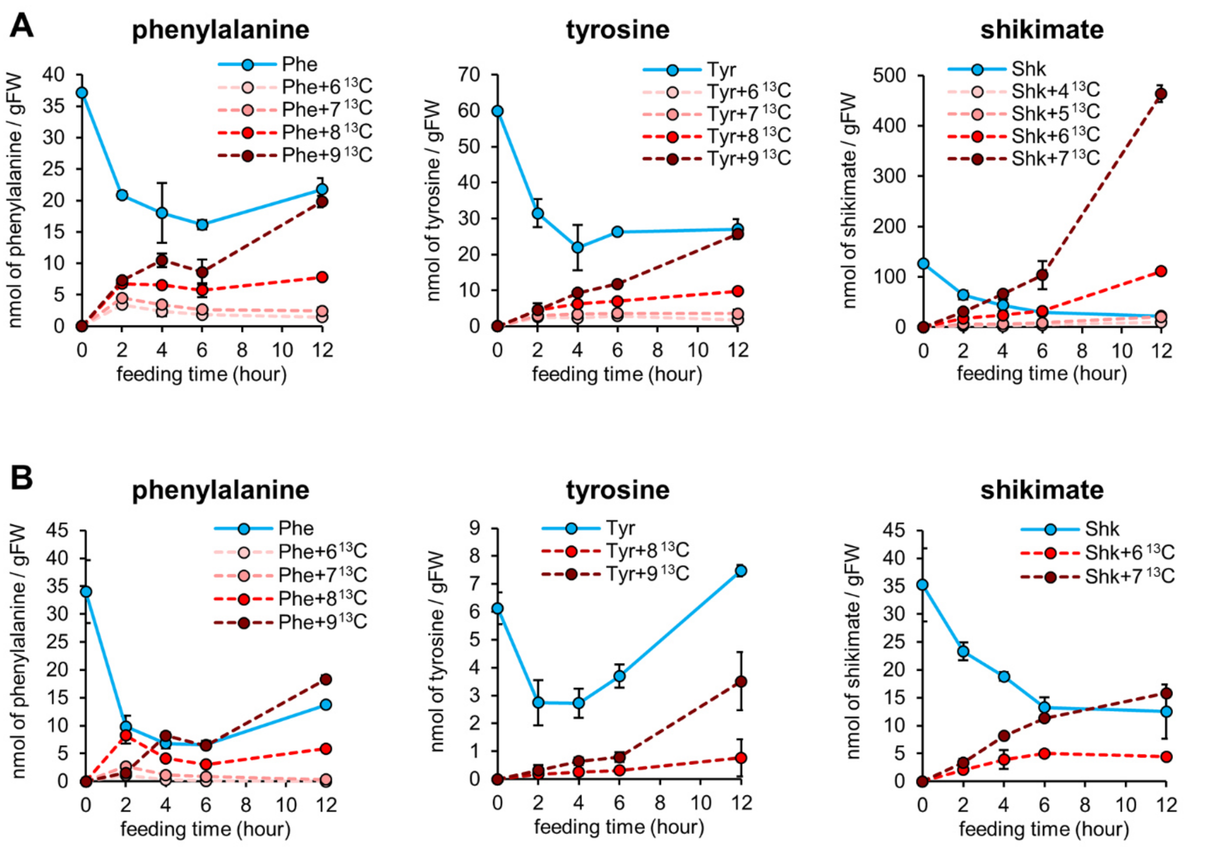

Supplemental Figure S1. ^{13}C-isotopologue abundance for phenylalanine (left), tyrosine (center) and shikimate (right) in **A)** *Arabidopsis thaliana* and **B)** *Brachypodium distachyon*. Solid lines in blue represent the concentration of unlabelled metabolite, dashed lines in red-pink tones correspond to different ^{13}C isotopologues. The other possible isotopologues were undetectable or only detectable as a trace, so were not represented in these graphs. +1-^{13}C isotopologues, which are naturally abundant, were not quantified for the study. Individual datapoints are the average of n = 2 biological replicates from two independent plants; error bars = *SD.*

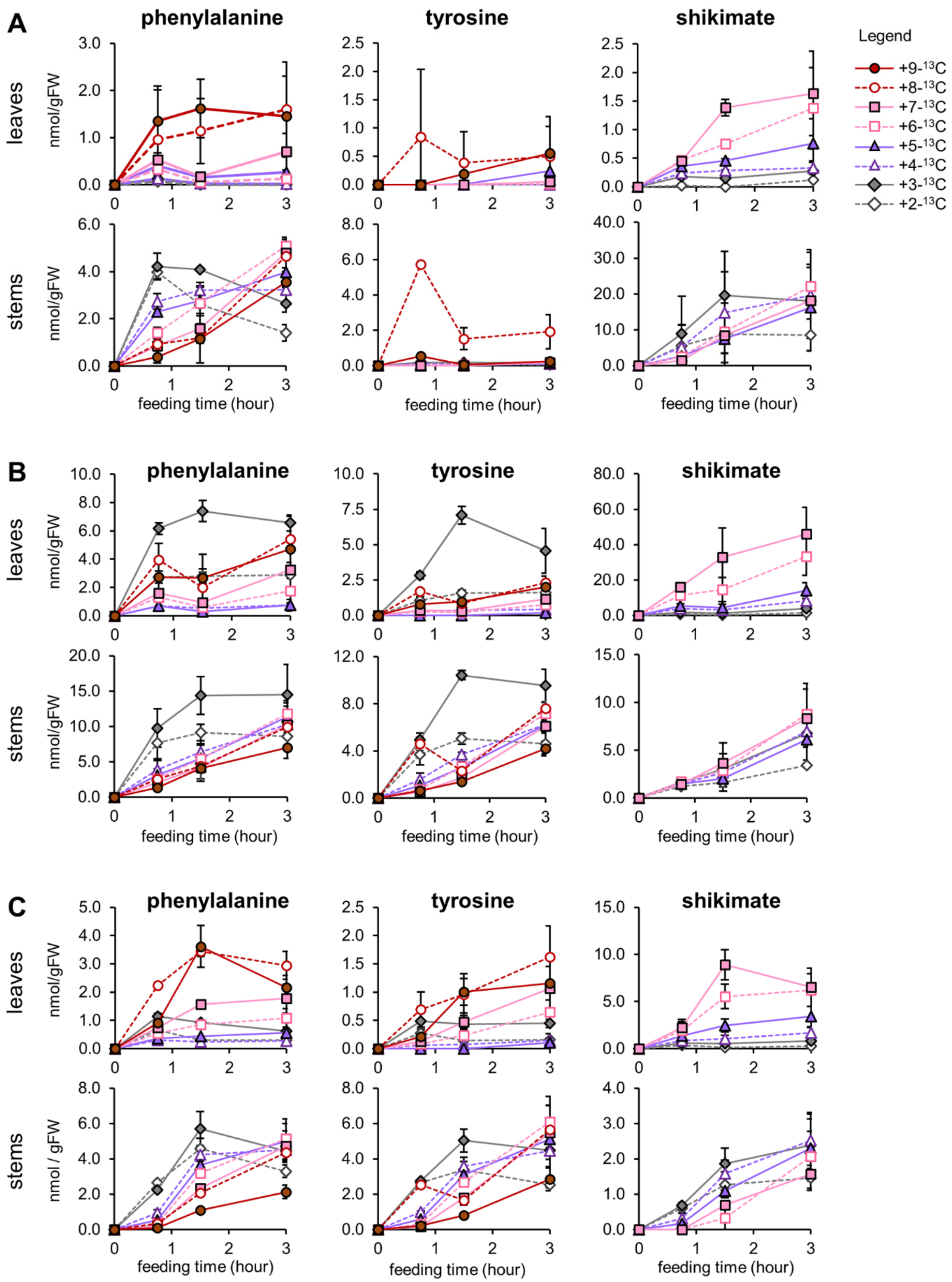
A
B
C
phenylalanine
tyrosine
shikimate
leaves
stems
nmol/gFW
nmol / gFW
feeding time (hour)
Legend
+9-13C
+8-13C
+7-13C
+6-13C
+5-13C
+4-13C
+3-13C
+2-13C

Supplemental Figure S2 (Previous page). ^{13}C-isotopologue abundance for phenylalanine (left column), tyrosine (center) and shikimate (right) in leaves and stems of **A)** *Arabidopsis thaliana,* **B)** *Brachypodium distachyon* and **C)** *Setaria viridis*. Isotopologues with only 1 ^{13}C atom, which are naturally present at high abundance, were not represented. Individual datapoints are the average of *n* = 2 biological replicates from two independent plants; error bars = *SD.*

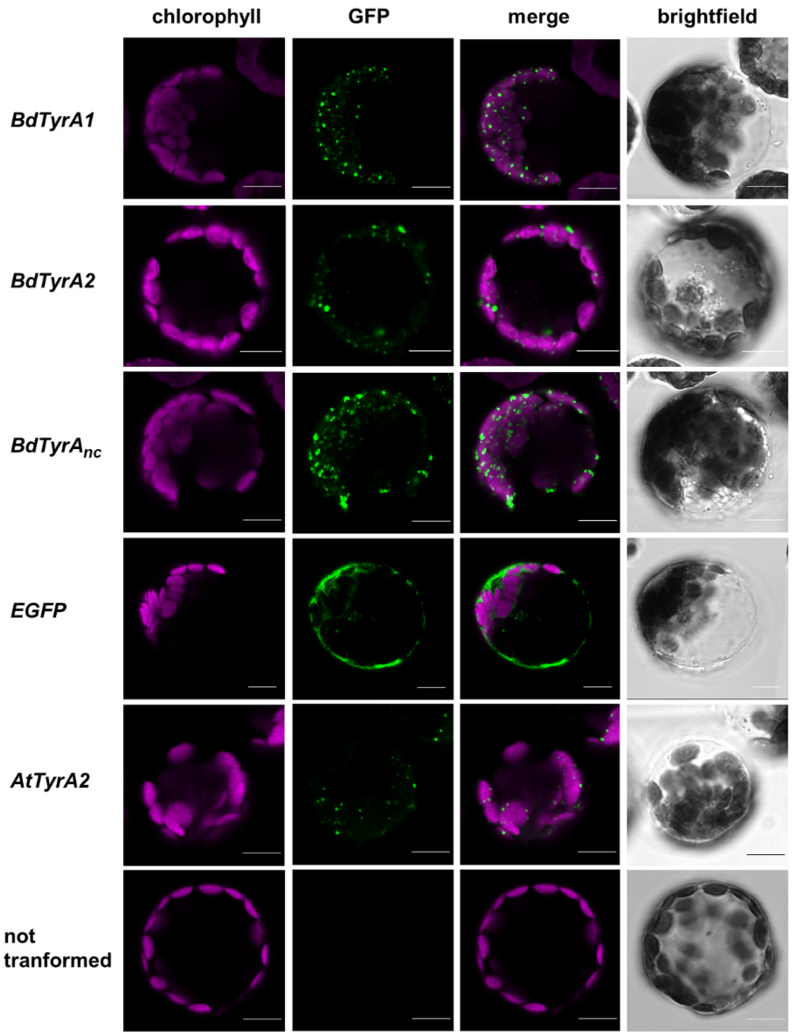

Supplemental Figure S3. Laser scan confocal microscopy of Arabidopsis protoplasts expressing the full lenght CDS of *Brachypodium distachyon*'s *BdTyrAs* fused to *EGFP*. *AtTyrA2-EGFP* was used as positive control for plastidial localization. Free *EGFP* was used as control for cytosolic localization. Scale bar = 10 µm.

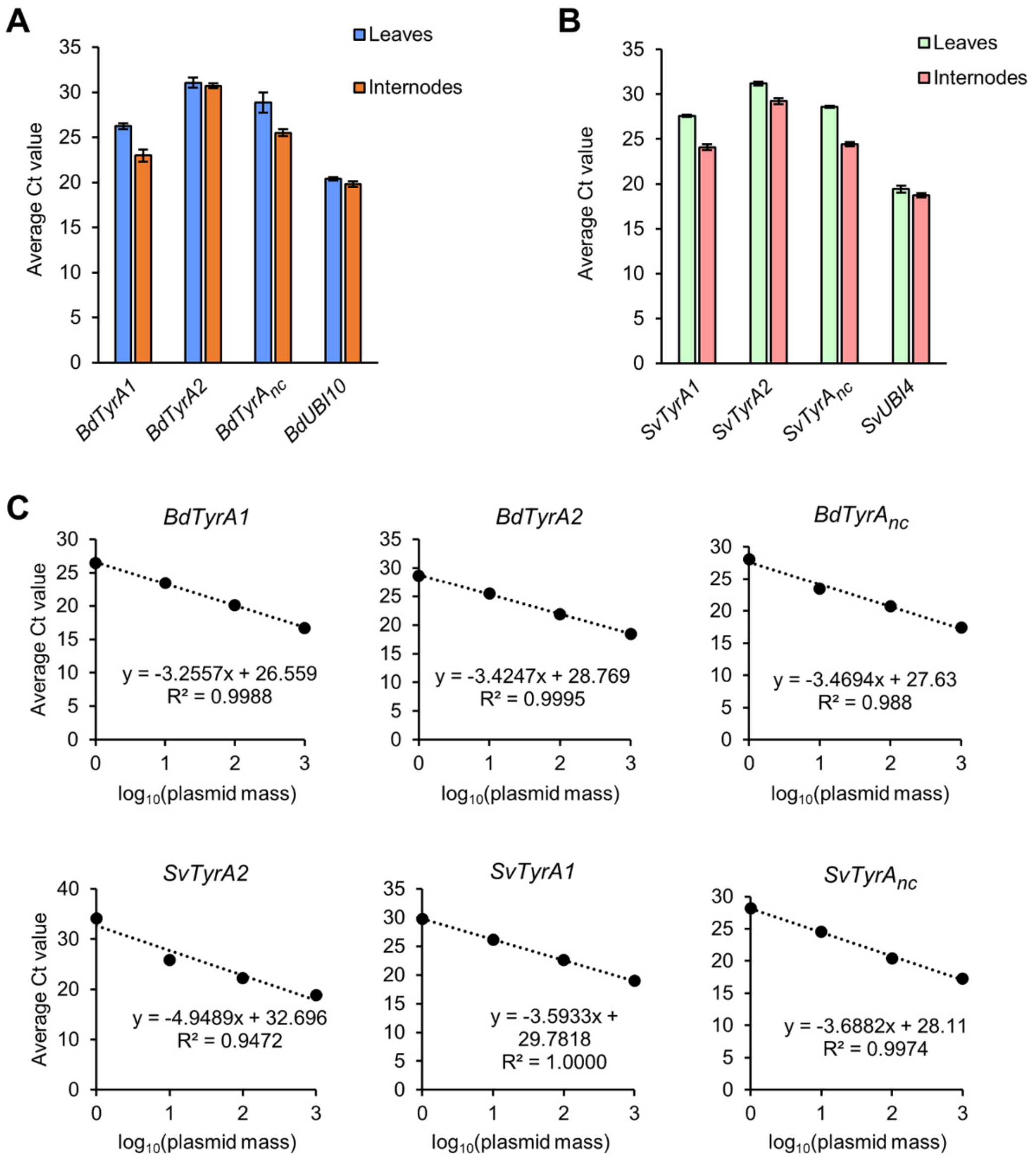

Supplemental Figure S4. Expression analysis of *TyrA* genes in *Brachypodium distachyon and Setaria viridis* leaves and developing internodes by RT-qPCR. A) Ct values in *Brachypodium distachyon* and **B)** *Setaria viridis.* Data presented as the average of n = 3 biological replicates coming from independent plants; error bars = *SD*. *BdUBI10* (*UBIQUITIN LIGASE 10*) and SvUBI4 (*UBIQUITIN LIGASE 4*) were used as reference genes. **C)** Standard curves were generated for each individual *TyrA* amplicon (*n* = 2 technical replicates).

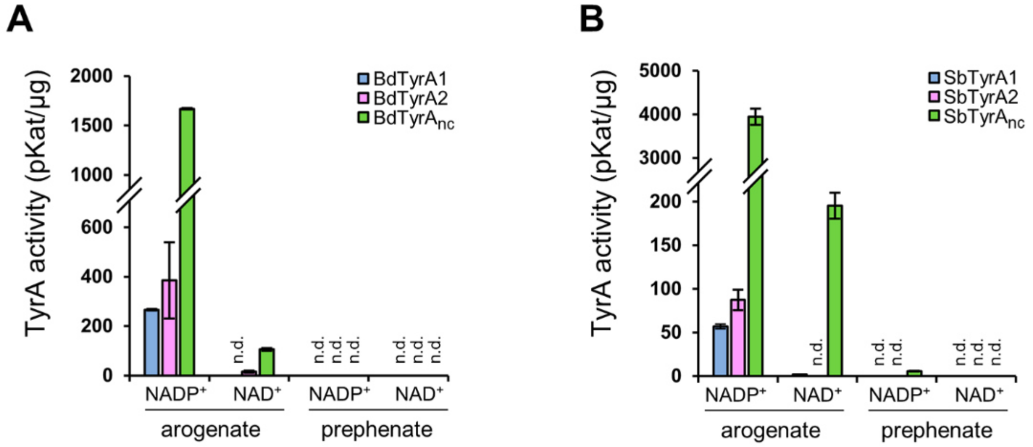

Supplemental Figure S5. Activity of pure recombinant TyrA enzymes towards alternative substrates (arogenate or prephenate) and electron acceptors (NAD^+ or $NADP^+$). **A)** Brachypodium TyrAs **B)** Sorghum TyrAs. Substrates and cofactors were tested at a concentration of 1 mM. Enzyme concentration was increased up to 10-times when using prephenate as substrate or NAD^+ as acceptor to increase assay's sensitivity. Data presented as the average of n = 3 independent assays; error bars = *SD*; n.d. = not detected.

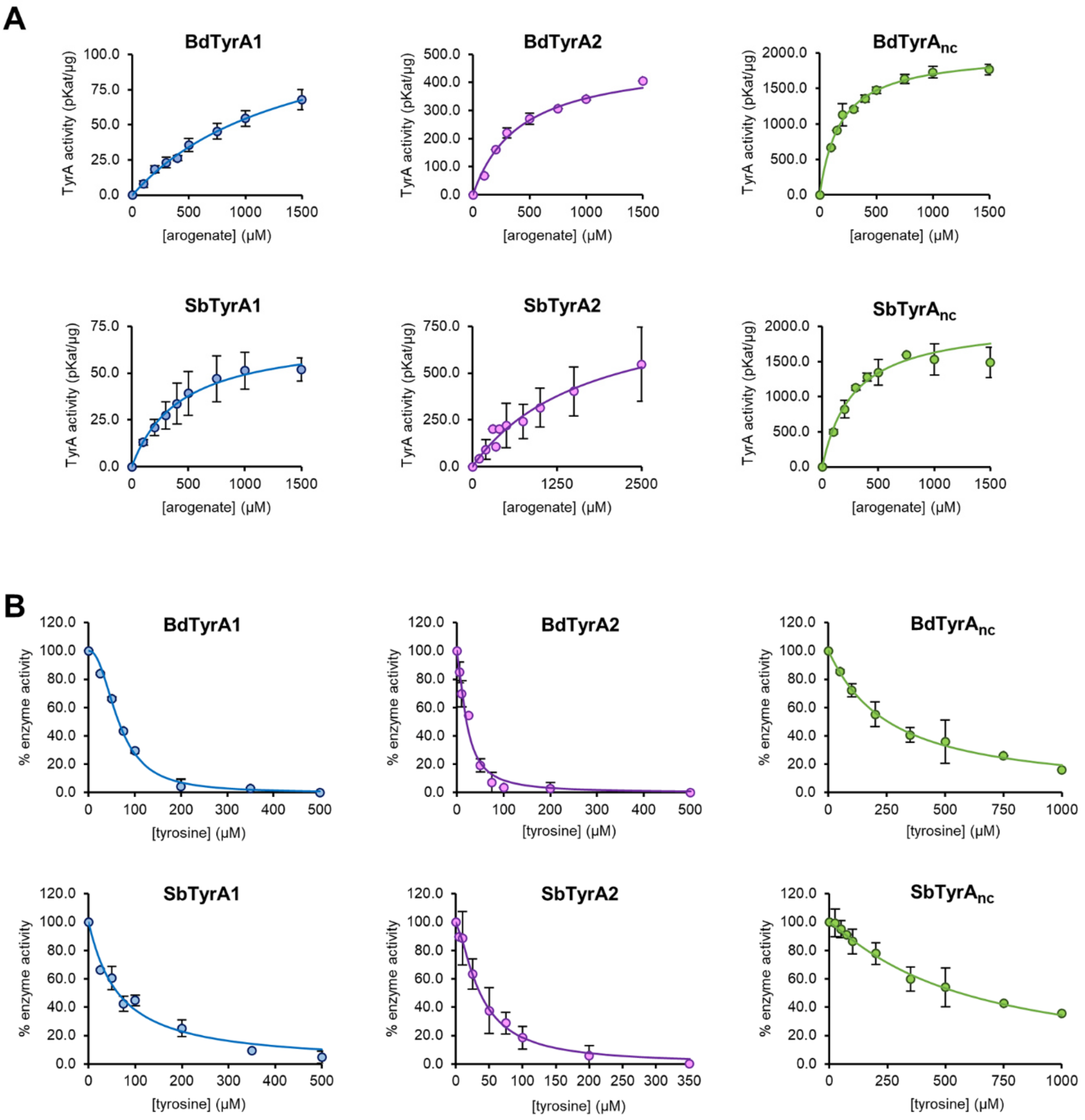

Supplemental Figure S6. *In vitro* kinetic characterization of recombinant grass TyrA enzymes. **A)** Michaelis-Menten plots corresponding to the kinetical parameters (K_m and V_{max}) shown in main Table 1. **B)** Tyrosine-inhibition plots used to calculate the IC_{50} value as shown in main Table 1. Individual points represent the average of 4 to 6 datapoints coming from at least two independent experiments conducted on different days using different batches of purified recombinant enzyme. Error bars = *SD*.

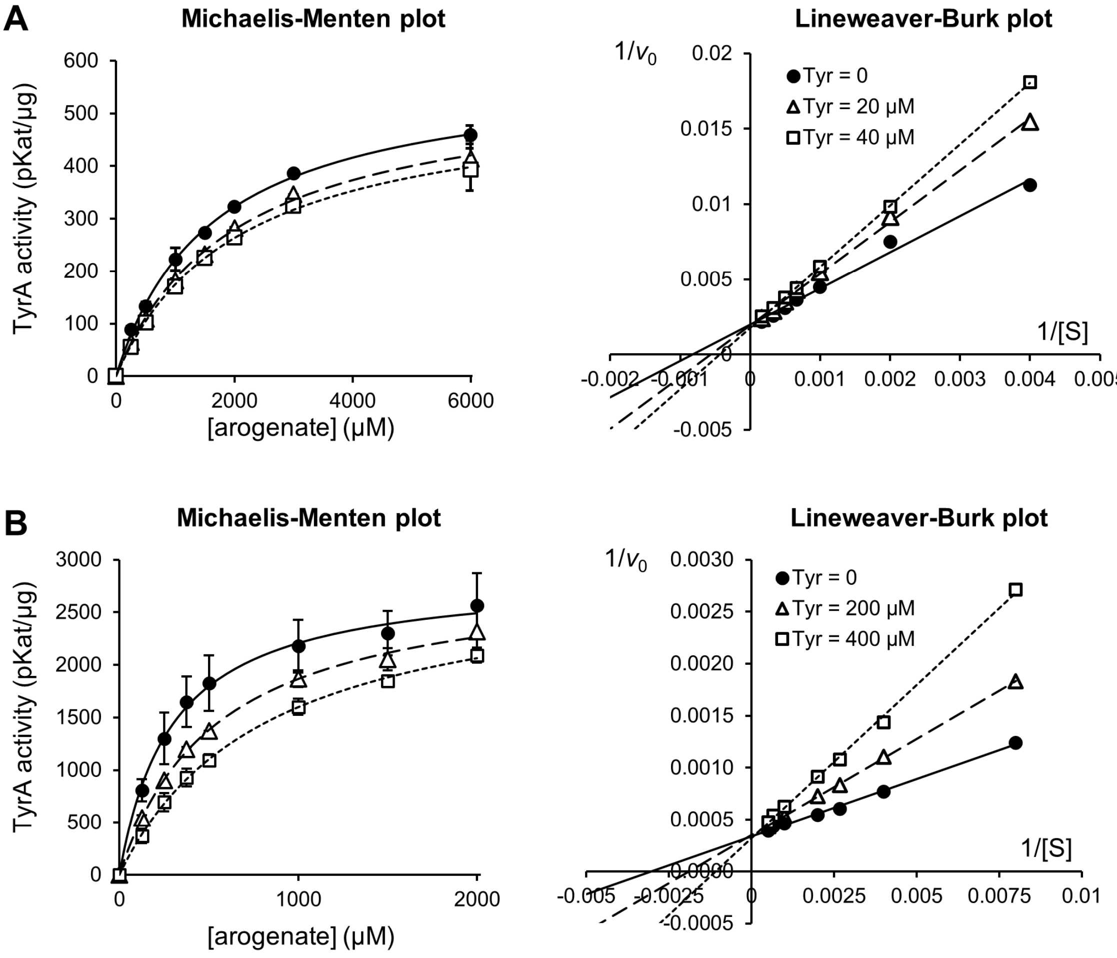

C

		Tyr = 0 µM	Tyr = 20 µM	Tyr = 40 µM
**BdTyrA1**	$K_{m\ app}$ **(µM arogenate)**	1623 ± 269	1876 ± 194	2162 ± 493
	$V_{max\ app}$ **(pKat/µg)**	586 ± 38	563 ± 64	551 ± 69

		Tyr = 0 µM	Tyr = 200 µM	Tyr = 400 µM
**BdTyrA$_{nc}$**	$K_{m\ app}$ **(µM arogenate)**	299 ± 38	549 ± 131	849 ± 248
	$V_{max\ app}$ **(pKat/µg)**	2861 ± 257	2894 ± 380	2946 ± 302

Supplemental Figure S7. Michaelis-Menten (left panel) and Lineweaver-Burk (right panel) plots corresponding to **A**) BdTyrA1 and **B**) BdTyrA$_{nc}$ assayed at two alternative concentrations of tyrosine (legends as in the right panels). **C)** Apparent K_m and V_{max} values as calculated from the data shown in A and B. Individual points represent the average of two technical replicates from the same experiment. Error bars = *SD*.

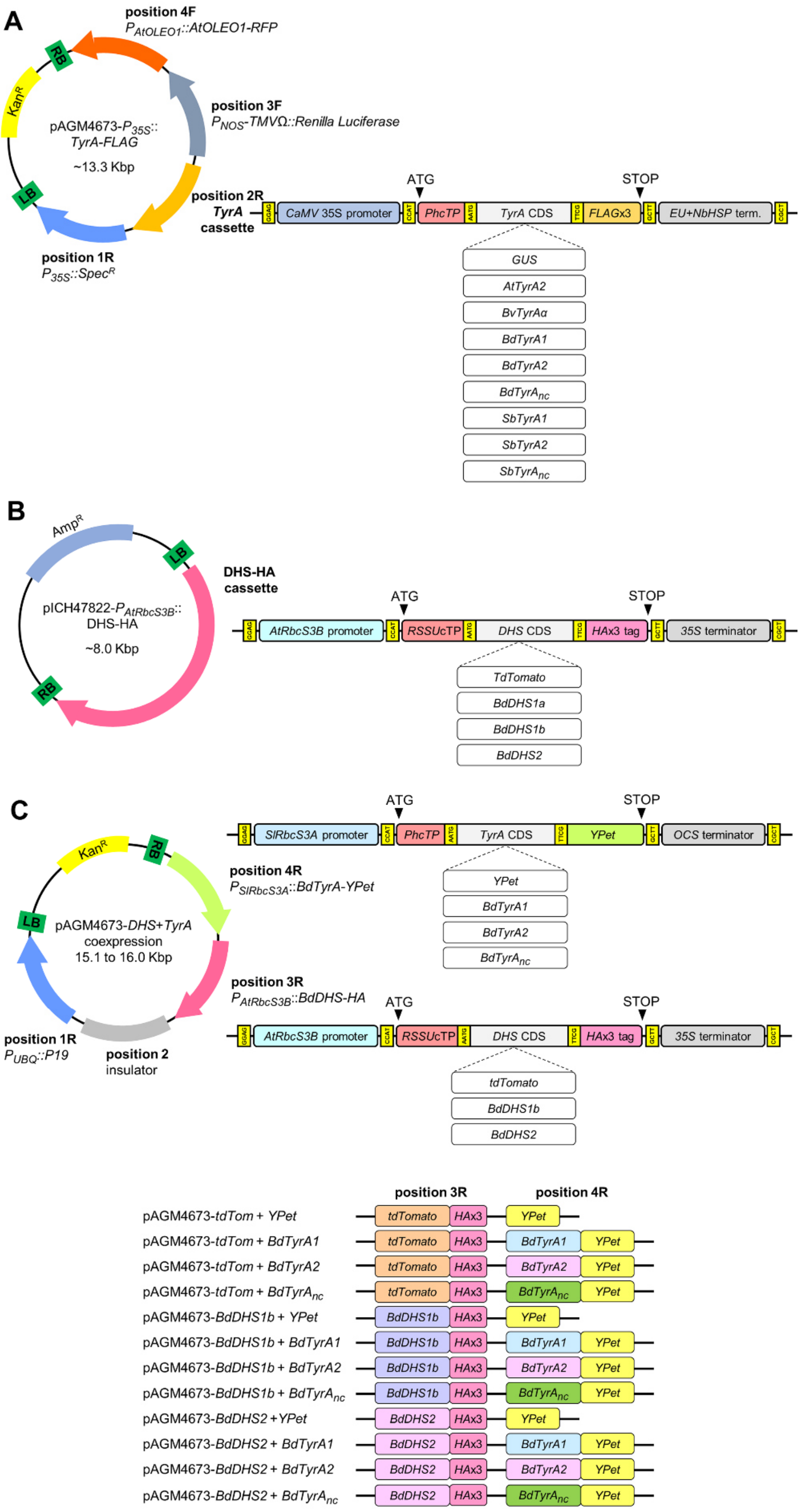
A
position 4F
PAtOLEO1::AtOLEO1-RFP
position 3F
PNOS-TMVΩ::Renilla Luciferase
pAGM4673-P35S::
TyrA-FLAG
~13.3 Kbp
KanR
RB
LB
position 2R
TyrA
cassette
position 1R
P35S::SpecR
ATG
STOP
CaMV 35S promoter
PhcTP
TyrA CDS
FLAGx3
EU+NbHSP term.
GUS
AtTyrA2
BvTyrAα
BdTyrA1
BdTyrA2
BdTyrAnc
SbTyrA1
SbTyrA2
SbTyrAnc
B
AmpR
LB
RB
pICH47822-PAtRbcS3B::
DHS-HA
~8.0 Kbp
DHS-HA
cassette
ATG
STOP
AtRbcS3B promoter
RSSUcTP
DHS CDS
HAx3 tag
35S terminator
TdTomato
BdDHS1a
BdDHS1b
BdDHS2
C
KanR
RB
LB
pAGM4673-DHS+TyrA
coexpression
15.1 to 16.0 Kbp
position 4R
PSlRbcS3A::BdTyrA-YPet
position 3R
PAtRbcS3B::BdDHS-HA
position 1R
PUBQ::P19
position 2
insulator
ATG
STOP
SlRbcS3A promoter
PhcTP
TyrA CDS
YPet
OCS terminator
YPet
BdTyrA1
BdTyrA2
BdTyrAnc
AtRbcS3B promoter
RSSUcTP
DHS CDS
HAx3 tag
35S terminator
tdTomato
BdDHS1b
BdDHS2
position 3R
position 4R
pAGM4673-tdTom + YPet
pAGM4673-tdTom + BdTyrA1
pAGM4673-tdTom + BdTyrA2
pAGM4673-tdTom + BdTyrAnc
pAGM4673-BdDHS1b + YPet
pAGM4673-BdDHS1b + BdTyrA1
pAGM4673-BdDHS1b + BdTyrA2
pAGM4673-BdDHS1b + BdTyrAnc
pAGM4673-BdDHS2 + YPet
pAGM4673-BdDHS2 + BdTyrA1
pAGM4673-BdDHS2 + BdTyrA2
pAGM4673-BdDHS2 + BdTyrAnc
tdTomato
BdDHS1b
BdDHS2
HAx3
YPet
BdTyrA1
BdTyrA2
BdTyrAnc

Supplemental Figure S8 (Previous page). Golden Gate constructs for transient expression of *TyrAs* and *DHSs* in *Nicotiana benthamiana* **A)** Level 2 vector for the expression of grass *TyrAs* under control of *CaMV* 35S promoter. *AtTyrA2*, *GUS* and *BvTyrAα* were used as controls. **B)** Level 1 vector for the expression of Brachypodium *DHSs* as HA tagged proteins, under control of Arabidopsis *AtRbcS3B* promoter. The fluorescent protein tdTomato was used as negative control. **C)** Level 2 assemblies of *TyrA-YPet* and *DHS-HA* level 1 vectors, with their corresponding controls. The *P19* repressor of the RNA silencing machinery was cloned in position 1 under control of the Arabidopsis' *UBIQUITIN LIGASE* promoter, as it was found to be critical to enhance the expression level of *BdDHS1b* and *BdDHS2*. Yellow boxes indicate Golden Gate overhangs used for the modules assembly. *Ph*cTP, plastid transit peptide of *Petunia* x *hybrida* 5-enol-pyruvyl-shikimate-3-phosphate synthase (Della-Cioppa et al., 1986). RSSUcTP, plastid transit peptide of Arabidopsis RuBisCO Small Subunit. *EU+NbHSP* terminator was based on Diamos AG, and Mason HS (2018). *NOS*, *Agrobacterium tumefaciens* nopaline synthase; *OCS*, *Agrobacterium tumefaciens* octopine synthase.

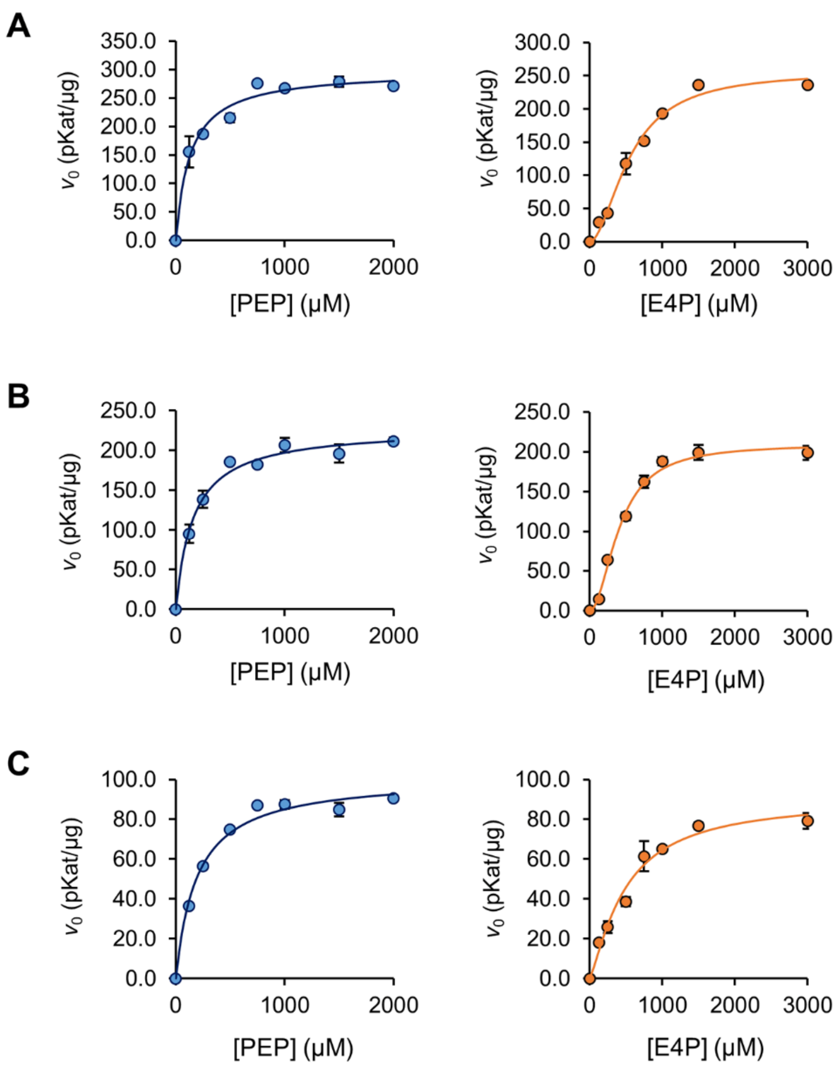

Supplemental Figure S9. Kinetic plots for phospho*enol*pyruvate (PEP, left) and erythrose 4-phosphate (E4P, right) for **A)** BdDHS1a **B)** BdDHS1b and **C)** BdDHS2. Datapoints points represent the average of at least two replicates from independent experiments using different preparations of purified recombinant enzyme. Error bars = *SD*.

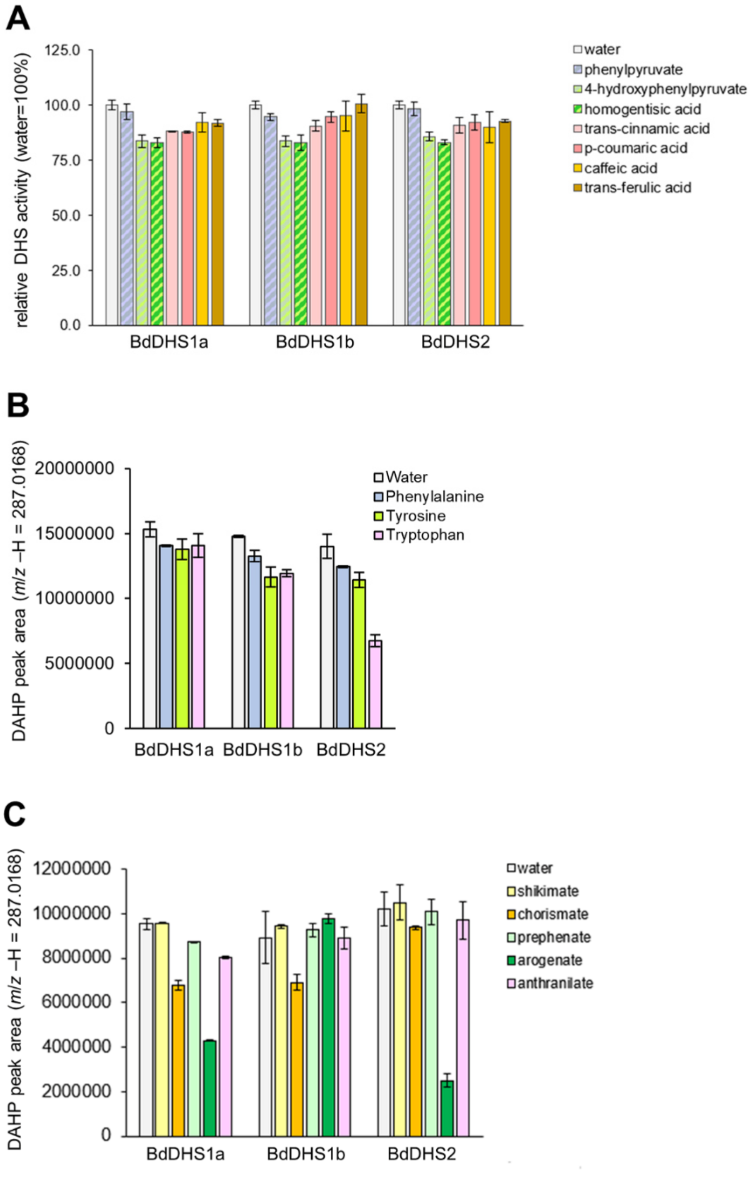

Supplemental Figure S10. Determination of the effect of aromatic amino acids and related compounds on recombinant Brachypodium DHSs. **A)** Modulation of Brachypodium DHSs by products of aromatic amino acid catabolism and lignin biosynthesis intermediates, determined by measuring phospho*enol*pyruvate consumption at 232 nm. **B)** Effect of aromatic amino acids and **C)** aromatic amino acid biosynthesis intermediates on Brachypodium DHSs, determined by UHPLC-MS quantification of DAHP production. All compounds were tested at a fixed concentration of 0.5 mM. Data presented as the average of *n* = 3 independent assays; error bars = *SD*

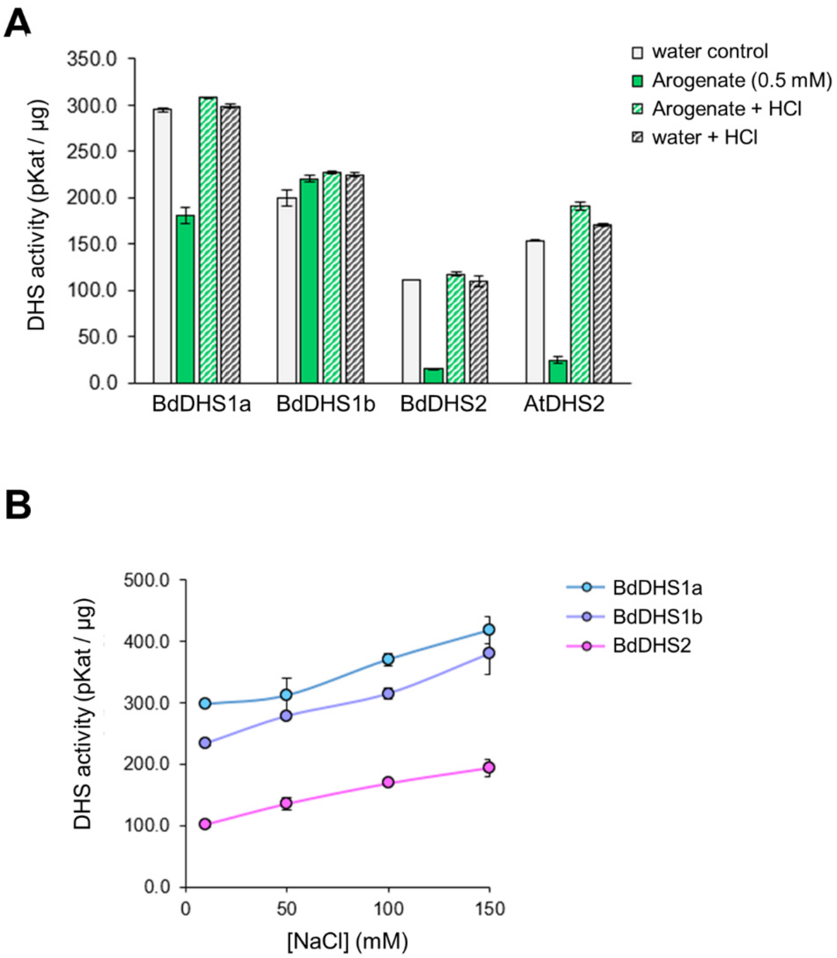

Supplemental Figure S11. Control experiments for the imapct of arogenate and excess salts over grass DHSs. **A)** Arogenate hydrolyzed to phenylalanine upon treatment with HCl 1N for 30 minutes (Zamir et al., 1980) does not have inhibitory effects on Brachypodium DHSs nor *Arabidopsis thaliana* AtDHS2, which was also previously reported to be inhibited by arogenate (Yokoyama et al., 2021). **B)** Effect of NaCl concentration on the activity of Brachypodium recombinant DHSs. Increasing concentrations of NaCl caused an increase in DHS activity of the different enzymes tested, which is the likely cause behind the increase in BdDHS1b activity when using high concentrations of arogenate, like in main Figure 4. Data presented as the average of *n* = 3 independent assays; error bars = *SD*

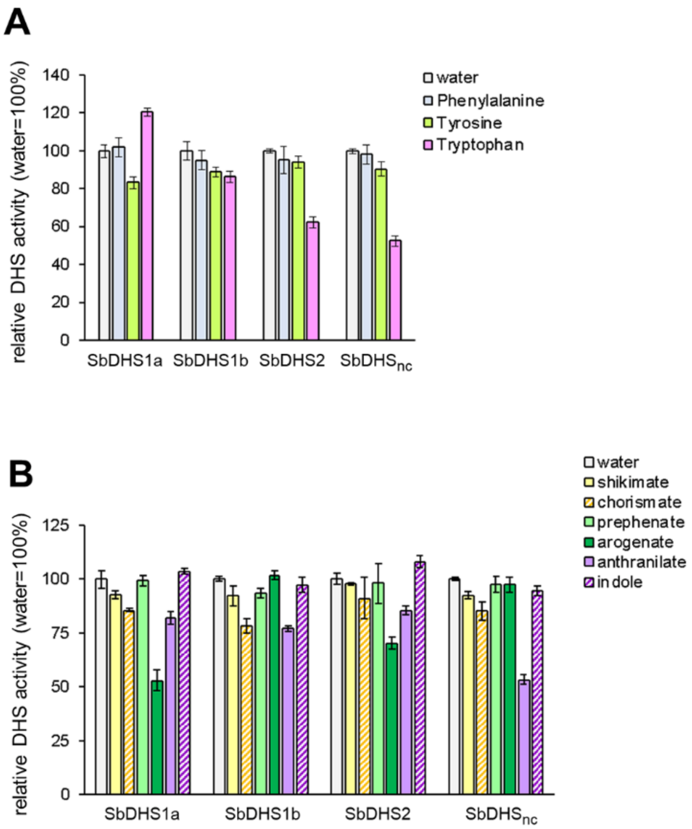

Supplemental Figure S12. Effect of aromatic amino acids and related compounds on recombinant Sorghum DHSs. **A)** Effect of aromatic amino acids on recombinant Sorghum DHSs, determined as phospho*enol*pyruvate consumption. **B)** Effect of aromatic amino acids intermediates on Sorghum DHSs, determined as phospho*enol*pyruvate consumption. All compounds were tested at a fixed concentration of 0.5 mM. Data presented as the average of n = 3 independent assays; error bars = *SD*

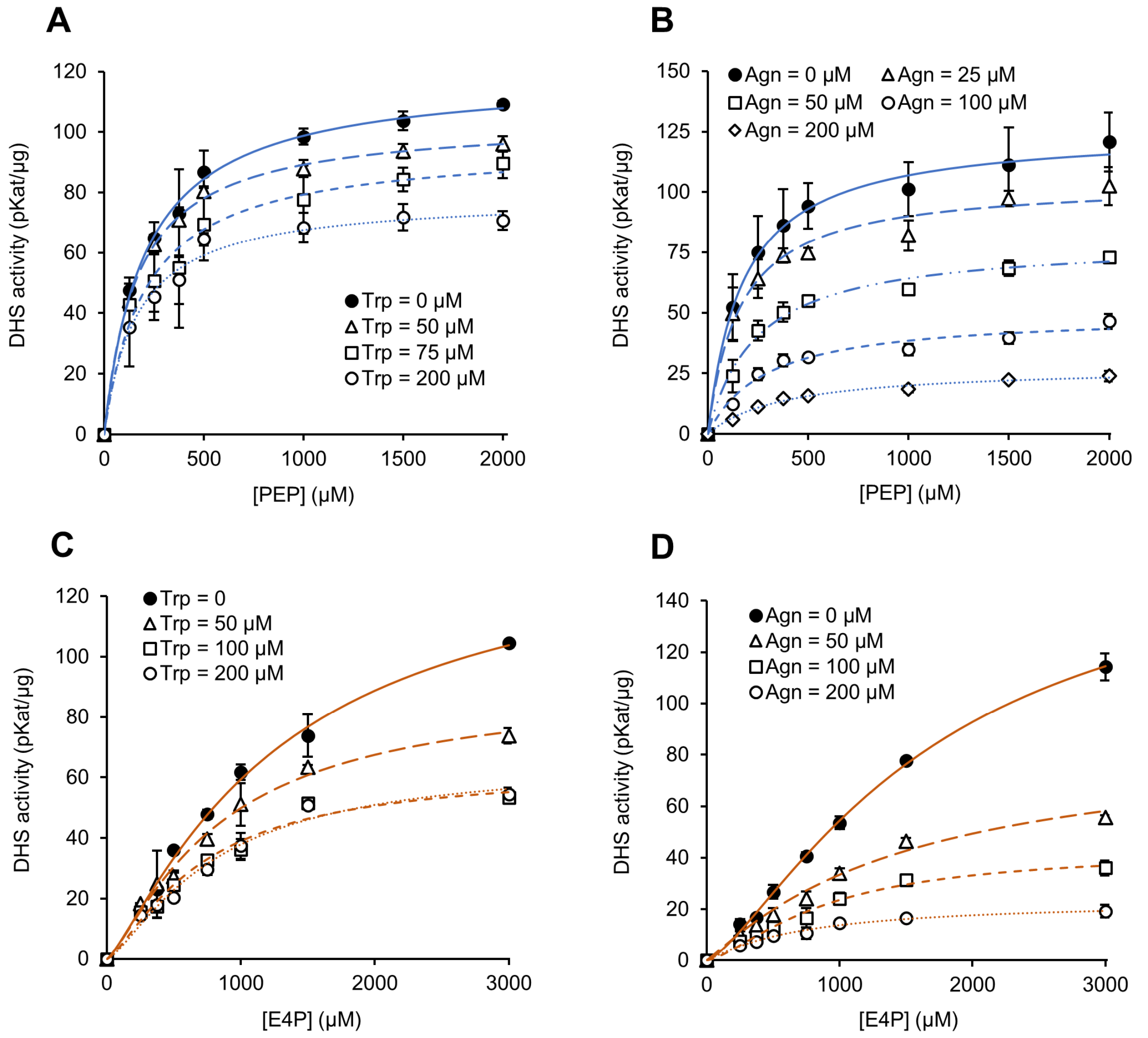

E

		Tryptophan (Trp)				Arogenate (Agn)				
		no Trp	50 µM	75 µM	200 µM	no Agn	25 µM	50 µM	100 µM	200 µM
**Phospho*enol*-pyruvate (PEP)**	**$V_{max\ app}$ (pKat/µg)**	119 ± 2	103 ± 2	97 ± 1	80 ± 4	126 ± 11	104 ± 2	80 ± 1	49 ± 5	28 ± 3
	**$K_{m\ app}$ (µM PEP)**	210 ± 65	166 ± 7	236 ± 131	186 ± 56	185 ± 72	161 ± 37	245 ± 60	288 ± 55	390 ± 68

		no Trp	50 µM	100 µM	200 µM	no Agn	50 µM	100 µM	200 µM
**Erythrose 4-phosphate (E4P)**	**$V_{max\ app}$ (pKat/µg)**	140 ± 9	90 ± 6	63 ± 6	66 ± 7	170 ± 30	69 ± 2	43 ± 5	22 ± 3
	**$K_{0.5\ app}$ (µM E4P)**	1293 ± 155	840 ± 117	694 ± 139	791 ± 166	1755 ± 445	1012 ± 17	870 ± 191	589 ± 173
	**Hill Coefficient**	1.2	1.3	1.3	1.3	1.4	1.4	1.5	1.3

Supplemental Figure S13. Michaelis-Menten plots for BdDHS2 at alternative concentrations of **A**) phospho*enol*pyruvate (PEP) and tryptophan, **B**) PEP and arogenate, **C**) erythrose 4-phosphate (E4P) and tryptophan, and **D**) E4P and arogenate. **E**) Apparent kinetical parameters calculated from the data shown in A to D. Hill equation was used to fit E4P kinetic data. Individual points represent the average of at least two to three technical replicates from the same experiment. Error bars = *SD*.

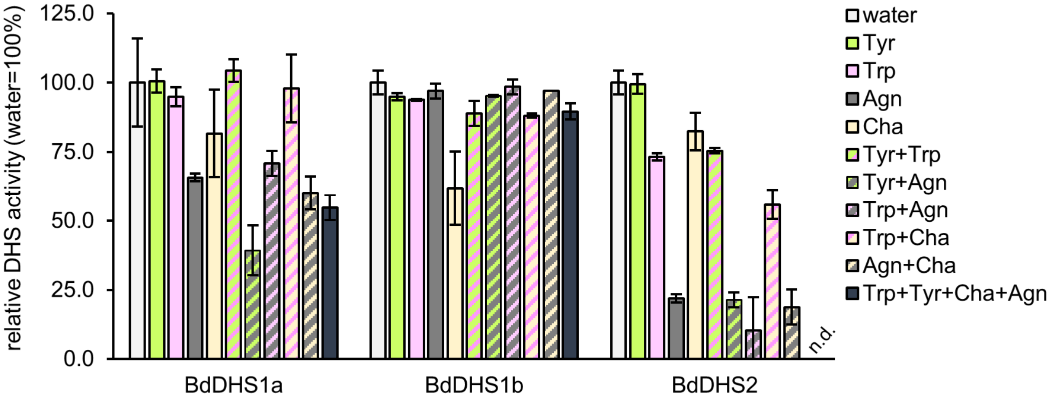

Supplemental Figure S14. Combined effect of tryptophan (Trp), tyrosine (Tyr), chorismate (Cha) and arogenate (Agn) on recombinant Brachypodium DHSs. All effectors were tested a a concentration of 0.15 mM each. Data presented as the average of *n* = 3 independent assays; error bars = *SD*; *n.d.* = not detected.

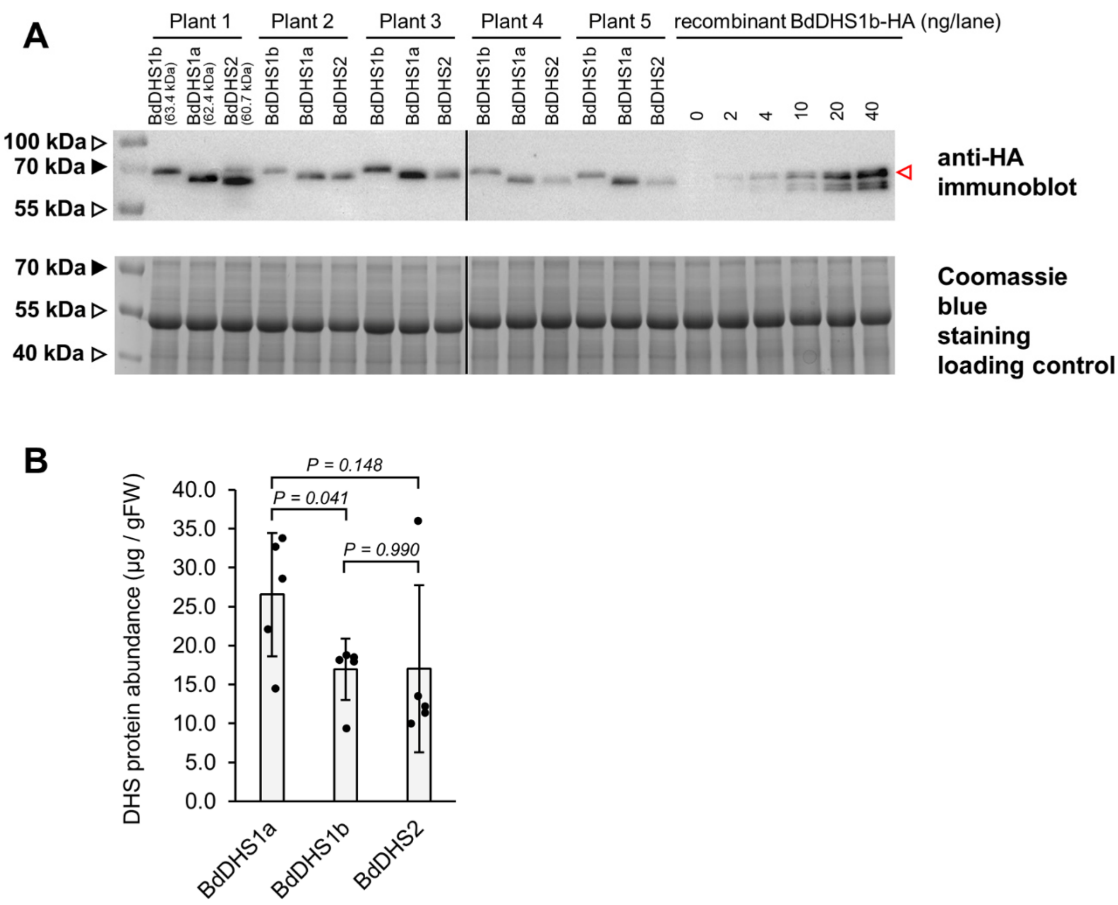

Supplemental Figure S15. Immunoblot quantification of Brachypodium DHSs fused to C-terminal HA tag upon transient expression in *Nicotiana benthamiana*. **A)** Anti-HA immunoblot (top) and Coomassie blue staining of total proteins as loading control (bottom) in samples coming from five independent plants. Expected size of the mature full lenght proteins: BdDHS1a, 62.4 kDa; BdDHS1b, 63.4 kDa; BdDHS2, 60.7 kDa. Calibration curve for the HA tag quantification was generated using pure recombinant BdDHS1b-HA protein mixed with total protein extract from a non-infiltrated leaf of *Nicotiana benthamiana*. Red arrow on the right indicates the expected size of the full lenght BdDHS1b-HA protein; bands of lower molecular weight in the BdDHS1b-HA standard are pressumably degradation of the HA tagged proteins in *E. coli*, and were not considered for quantitative purposes. All immunoblot images were not-saturated and all shown membranes were exposed simultaneously. Vertical line separate independent membranes/gels. **B)** DHS protein abundance by gFW of plant sample, based on anti-HA immunoblot signal. *P* values according to Students' *t*-test (two-sided) for two samples with equal variance. Data presented as the average of *n* = 5 biological simples from independent plants; error bars = *SD*.
