# Supplementary material for: Coordinated regulation of the entry and exit steps of aromatic amino acid biosynthesis supports the dual lignin pathway in grasses
Source: Nat Commun. 2023 Nov 9;14:7242. doi: 10.1038/s41467-023-42587-7 (PMC10636026; doi:10.1038/s41467-023-42587-7)
Supplement: Supplementary file 3 — Description of Additional Supplementary Files [file 41467_2023_42587_MOESM3_ESM.pdf]

Description of Additional Supplementary Files

Supplementary Data 1

List of PTAL (Bradi3g49250) co-expressed genes in Brachypodium distachyon, available at http://aranet.mpimp-golm.mpg.de/

Supplementary Data 2 List of primers used in this study
