# Supplementary material for: Coordinated regulation of the entry and exit steps of aromatic amino acid biosynthesis supports the dual lignin pathway in grasses
Source: Nat Commun. 2023 Nov 9;14:7242. doi: 10.1038/s41467-023-42587-7 (PMC10636026; doi:10.1038/s41467-023-42587-7)
Supplement: Supplementary file 4 — Supplementary Data 1 [file 41467_2023_42587_MOESM4_ESM.pdf]

Supplemental Table S1. List of genes coexpressed with *BdPTAL* . *BdPTAL, BdTyrA1* and *BdDHS1b* are highlighted in salmon. Data obtained from PlaNet at http://aranet.mpimp-golm.mpg.de/ (Comparative phylogenomic analysis of gene co-expression networks reveals evolution of functional modules in plants. Ruprecht et al., 2016).

Probeset/other ID	GeneID	Description	Label(s) present
Bradi3g49250.2	bradi3g49250	phenylalanine ammonia-lyase, putative, expressed (*BdPTAL*)	PAL ORTHO000087 HOM000613
Bradi1g28490.1	bradi1g28490	fiber protein Fb34, putative, expressed	DUF1218 ORTHO002392 HOM000854
Bradi5g12460.1	bradi5g12460	uncharacterized Cys-rich domain containing protein, putative, expressed	ORTHO001933 HOM000387
Bradi4g01370.1	bradi4g01370	DNA binding protein, putative, expressed	Branch ORTHO007270 HOM000186
Bradi3g38950.1	bradi3g38950	methyladenine glycosylase, putative, expressed	Adenine_glyco ORTHO009146 HOM000675
Bradi2g18447.1	bradi2g18447	sulfotransferase domain containing protein, expressed	ORTHO017557 HOM000207
Bradi2g12370.1	bradi2g12370	GDSL-like lipase/acylhydrolase, putative, expressed	Lipase_GDSL ORTHO022049 HOM000086
Bradi2g26770.1	bradi2g26770	annexin, putative, expressed	Annexin ORTHO009375 HOM000320
Bradi4g01717.1	bradi4g01717	galactosyltransferase family protein, putative, expressed	ORTHO000137 HOM000468
Bradi2g17067.1	bradi2g17067	auxin-responsive protein, putative, expressed	ORTHO022083 HOM000285
Bradi1g10150.1	bradi1g10150	tubulin/FtsZ domain containing protein, putative, expressed	Tubulin Tubulin_C ORTHO000065 HOM000128
Bradi3g13237.1	bradi3g13237	transferase family protein, putative, expressed	ORTHO000849 HOM000359
Bradi2g48267.1	bradi2g48267	alliin lyase precursor, putative, expressed	ORTHO001656 HOM000677
Bradi3g04920.1	bradi3g04920	auxin response factor 6, putative, expressed	AUX_IAA Auxin_resp B3 ORTHO000238 HOM000105
Bradi4g01200.2	bradi4g01200	5-methyltetrahydropteroyltriglutamate--homocysteine methyltransferase, putative, expressed	Meth_synt_1 Meth_synt_2 ORTHO000530 HOM001867
Bradi4g21240.1	bradi4g21240	plant-specific domain TIGR01627 family protein, expressed	DUF579 HOM000646
Bradi2g43690.1	bradi2g43690	oxidoreductase, aldo/keto reductase family protein, putative, expressed	Aldo_ket_red ORTHO019317 HOM000250
Bradi1g03947.1	bradi1g03947	leaf senescence related protein, putative, expressed	ORTHO021320 HOM000050
Bradi5g02460.1	bradi5g02460	cytochrome P450 93A2, putative, expressed	ORTHO019696 HOM000005
Bradi3g33070.1	bradi3g33070	expressed protein	DUF566 ORTHO022740 HOM000545
Bradi3g59810.1	bradi3g59810	endoglucanase, putative, expressed	Glyco_hydro_9 ORTHO000312 HOM000137
Bradi2g46197.1	bradi2g46197	no apical meristem protein, putative, expressed	ORTHO008852 HOM000659
Bradi3g05670.1	bradi3g05670	STRUBBELIG-RECEPTOR FAMILY 3 precursor, putative, expressed	Pkinase Pkinase_Tyr ORTHO014618 HOM000004
Bradi1g06090.1	bradi1g06090	ubiquitin-conjugating enzyme, putative, expressed	UQ_con HOM000068
Bradi3g52790.1	bradi3g52790	basic helix-loop-helix, putative, expressed	HLH ORTHO022931 HOM000075
Bradi3g16670.1	bradi3g16670	peptide transporter PTR2, putative, expressed	PTR2 ORTHO001640 HOM000031
Bradi3g05010.1	bradi3g05010	tubulin/FtsZ domain containing protein, putative, expressed	Misat_Myo_SegII Tubulin Tubulin_C ORTHO000021 HOM000128
Bradi1g66720.1	bradi1g66720	laccase precursor protein, putative, expressed	Cu-oxidase Cu-oxidase_2 Cu-oxidase_3 ORTHO000023 HOM000096
Bradi1g03940.1	bradi1g03940	leaf senescence related protein, putative, expressed	DUF231 ORTHO006504 HOM000050
Bradi3g11470.4	bradi3g11470	ras-related protein, putative, expressed	Ras ORTHO032541 HOM000032
Bradi2g23530.1	bradi2g23530	homeodomain protein, putative, expressed	Coprinus_mating Homeobox POX ORTHO019268 HOM000277

Bradi2g34240.1	bradi2g34240	CESA1 - cellulose synthase, expressed	Cellulose_synt Glycos_transf_2 ORTHO000003 HOM000082
Bradi1g21410.1	bradi1g21410	expressed protein	ORTHO008419 HOM001687
Bradi1g03880.1	bradi1g03880	AP2 domain containing protein, expressed	AP2 ORTHO021318 HOM000119
Bradi1g58997.1	bradi1g58997	peroxidase precursor, putative, expressed	ORTHO003579 HOM000012
Bradi1g33230.1	bradi1g33230	expressed protein	DUF3511 ORTHO007368 HOM000519
Bradi4g40400.1	bradi4g40400	plant-specific domain TIGR01627 family protein, expressed	ORTHO007686 HOM000646
Bradi2g61070.1	bradi2g61070	NADPH quinone oxidoreductase, putative, expressed	FMN_red ORTHO002757 HOM002281
Bradi3g30670.1	bradi3g30670	dehydration response related protein, putative, expressed	DUF248 ORTHO000054 HOM000110
Bradi1g13910.1	bradi1g13910	START domain containing protein, expressed	Homeobox MEKHLA START bZIP_1 ORTHO000117 HOM000805
Bradi1g60852.1	bradi1g60852	expressed protein	ORTHO021782 HOM001687
Bradi4g16560.1	bradi4g16560	cytochrome P450, putative, expressed	p450 ORTHO019002 HOM000005
Bradi1g00710.1	bradi1g00710	expressed protein	DUF547 ORTHO010994 HOM000340
Bradi1g26510.1	bradi1g26510	glucan endo-1,3-beta-glucosidase precursor, putative, expressed	Glyco_hydro_17 X8 ORTHO010578 HOM000021
Bradi4g34300.1	bradi4g34300	membrane protein, putative, expressed	Cytochrom_B561 Cytochrom_C_asm DUF568 ORTHO003253 HOM000285
Bradi2g54940.1	bradi2g54940	homeodomain protein, putative, expressed	Coprinus_mating Homeobox POX ORTHO006589 HOM000277
Bradi3g58560.1	bradi3g58560	plastocyanin-like domain containing protein, putative, expressed	Cu_bind_like ORTHO032695 HOM000055
Bradi1g59820.1	bradi1g59820	legume lectins beta domain containing protein, putative, expressed	Lectin_legB ORTHO021766 HOM015280
Bradi2g46810.1	bradi2g46810	expressed protein	ORTHO032453 HOM001415
Bradi1g72430.1	bradi1g72430	STRUBBELIG-RECEPTOR FAMILY 6 precursor, putative, expressed	Pkinase Pkinase_Tyr ORTHO001429 HOM000004
Bradi2g21860.1	bradi2g21860	NADH-cytochrome b5 reductase, putative, expressed	FAD_binding_6 NAD_binding_1 ORTHO001038 HOM000208
Bradi2g23370.1	bradi2g23370	laccase precursor protein, putative, expressed	Cu-oxidase Cu-oxidase_2 Cu-oxidase_3 ORTHO000023 HOM000096
Bradi1g34670.1	bradi1g34670	glycosyltransferase, putative, expressed	DUF563 ORTHO021591 HOM000406
Bradi3g00377.1	bradi3g00377	rhodanese-like domain containing protein, putative, expressed	ORTHO013010 HOM001275
Bradi1g71680.2	bradi1g71680	LTPL69 - Protease inhibitor/seed storage/LTP family protein precursor, expressed	Tryp_alpha_amyl ORTHO002853 HOM000917
Bradi4g24650.1	bradi4g24650	abscisic stress-ripening, putative, expressed	ABA_WDS ORTHO032784 HOM010393
Bradi4g35477.1	bradi4g35477	bile acid sodium symporter family protein, putative, expressed	ORTHO010297 HOM000916
Bradi3g39170.1	bradi3g39170	harpin-induced protein 1 domain containing protein, expressed	LEA_2 ORTHO009594 HOM002508
Bradi4g31130.1	bradi4g31130	ferric reductase, putative, expressed	FAD_binding_8 Ferric_reduct NADPH_Ox NAD_binding_6 ORTHO000035 HOM000327
Bradi2g17982.1	bradi2g17982	myb-like DNA-binding domain containing protein, putative, expressed	HOM000007
Bradi5g19960.1	bradi5g19960	RING-H2 finger protein ATL5G, putative, expressed	ORTHO023430 HOM000013
Bradi1g68280.1	bradi1g68280	actin-depolymerizing factor, putative, expressed	Cofilin_ADF ORTHO011339 HOM000392
Bradi1g09460.1	bradi1g09460	endoglucanase, putative, expressed	Glyco_hydro_9 ORTHO000460 HOM000137
Bradi1g54250.1	bradi1g54250	CESA8 - cellulose synthase, expressed	Cellulose_synt Glycos_transf_2 ORTHO000003 HOM000082
Bradi5g08907.1	bradi5g08907	heparan-alpha-glucosaminide N-acetyltransferase, putative, expressed	ORTHO004670 HOM000992
Bradi2g26760.1	bradi2g26760	annexin, putative, expressed	Annexin ORTHO008631 HOM000320

Bradi2g16560.1	bradi2g16560	fasciclin domain containing protein, expressed	Fasciclin ORTHO026432 HOM000419
Bradi3g16530.1	bradi3g16530	O-methyltransferase, putative, expressed	Dimerisation Methyltransf_2 ORTHO002068 HOM000097
Bradi1g14110.1	bradi1g14110	expressed protein	DUF828 PH_2 ORTHO005020 HOM000922
Bradi2g54680.1	bradi2g54680	laccase precursor protein, putative, expressed	Cu-oxidase Cu-oxidase_2 Cu-oxidase_3 ORTHO000023 HOM000096
Bradi1g64830.1	bradi1g64830	glycosyl transferase 8 domain containing protein, putative, expressed	Glyco_transf_8 ORTHO005871 HOM000191
Bradi2g51990.1	bradi2g51990	bHelix-loop-helix transcription factor, putative, expressed	HLH ORTHO026569 HOM000356
Bradi3g40850.1	bradi3g40850	ras-related protein, putative, expressed	Ras ORTHO001518 HOM000032
Bradi1g06290.1	bradi1g06290	fasciclin domain containing protein, expressed	Fasciclin ORTHO003396 HOM002140
Bradi5g14720.1	bradi5g14720	transferase family protein, putative, expressed	ORTHO001336 HOM000062
Bradi3g13420.1	bradi3g13420	esterase, putative, expressed	Abhydrolase_1 DUF1234 Thioesterase ORTHO008079 HOM000203
Bradi1g35477.1	bradi1g35477	STRUBBELIG-RECEPTOR FAMILY 7 precursor, putative, expressed	ORTHO002829 HOM000004
Bradi2g47590.1	bradi2g47590	MYB family transcription factor, putative, expressed	Actin Myb_DNA-binding ORTHO022335 HOM000007
Bradi5g10210.1	bradi5g10210	OsSub41 - Putative Subtilisin homologue, expressed	ORTHO004169 HOM000020
Bradi4g21220.1	bradi4g21220	auxin-induced protein 5NG4, putative, expressed	EamA ORTHO003362 HOM000059
Bradi2g60310.1	bradi2g60310	zinc finger protein, putative, expressed	ORTHO007281 HOM002119
Bradi5g25767.1	bradi5g25767	auxin response factor, putative, expressed	ORTHO000238 HOM000105
Bradi1g25117.1	bradi1g25117	CSLF2 - cellulose synthase-like family F; beta1,3;1,4 glucan synthase, expressed	ORTHO000003 HOM000082
Bradi1g34790.1	bradi1g34790	arogenate dehydrogenase 1, chloroplast precursor, putative, expressed (*BdTyrA1*)	F420_oxidored PDH ORTHO001219 HOM000354
Bradi1g50280.1	bradi1g50280	expressed protein	ORTHO012178 HOM006709
Bradi3g28350.1	bradi3g28350	CESA7 - cellulose synthase, expressed	Cellulose_synt Glycos_transf_2 ORTHO000003 HOM000082
Bradi2g56397.1	bradi2g56397	proton-dependent oligopeptide transport, putative, expressed	ORTHO032480 HOM000031
Bradi2g55340.1	bradi2g55340	transmembrane amino acid transporter protein, putative, expressed	Aa_trans ORTHO000128 HOM000823
Bradi4g36240.1	bradi4g36240	endoglucanase, putative, expressed	Glyco_hydro_9 ORTHO001222 HOM000137
Bradi1g60750.1	bradi1g60750	phospho-2-dehydro-3-deoxyheptonate aldolase, chloroplast precursor, putative, expressed (*BdDHS1b*)	DAHP_synth_2 ORTHO000246 HOM000967
Bradi1g01870.1	bradi1g01870	pirin, putative, expressed	Pirin Pirin_C ORTHO005793 HOM001456
Bradi3g34615.2	bradi3g34615	OsSCP22 - Putative Serine Carboxypeptidase homologue, expressed	ORTHO022664 HOM000054
Bradi4g28260.1	bradi4g28260	hydroxyproline-rich glycoprotein family protein, putative, expressed	HOM089020
Bradi4g22250.1	bradi4g22250	dirigent, putative, expressed	Dirigent ORTHO016006 HOM000170
Bradi3g06480.1	bradi3g06480	dehydrogenase, putative, expressed	ADH_N ADH_zinc_N ORTHO009302 HOM000222
Bradi1g11090.1	bradi1g11090	GRAS family transcription factor domain containing protein, expressed	DELLA GRAS ORTHO000915 HOM000042
Bradi2g21300.1	bradi2g21300	cytochrome P450, putative, expressed	p450 ORTHO001576 HOM000005
Bradi1g47767.1	bradi1g47767	inorganic H+ pyrophosphatase, putative, expressed	ORTHO000186 HOM000354
Bradi1g33160.1	bradi1g33160	auxin response factor 18, putative, expressed	AUX_IAA Auxin_resp B3 ORTHO000307 HOM000105
Bradi5g25090.1	bradi5g25090	IQ calmodulin-binding motif family protein, putative, expressed	ORTHO019770 HOM005563
Bradi1g29560.1	bradi1g29560	pollen signalling protein with adenylyl cyclase activity, putative, expressed	NB-ARC ORTHO021547 HOM000009

Bradi3g51387.1	bradi3g51387	aquaporin protein, putative, expressed	HOM000085
Bradi4g34040.1	bradi4g34040	CHIT13 - Chitinase family protein precursor, expressed	Glyco_hydro_19 ORTHO004207 HOM000272
Bradi3g45160.1	bradi3g45160	harpin-induced protein 1 domain containing protein, expressed	LEA_2 ORTHO022851 HOM000572
Bradi2g56970.1	bradi2g56970	amino acid transporter, putative, expressed	Aa_trans Trp_Tyr_perm ORTHO000124 HOM000159
Bradi4g44530.1	bradi4g44530	peroxidase precursor, putative, expressed	ORTHO001479 HOM000012
Bradi3g36887.1	bradi3g36887	cinnamoyl-CoA reductase, putative, expressed	ORTHO002362 HOM000069
Bradi4g13670.1	bradi4g13670	patatin, putative, expressed	Patatin ORTHO015349 HOM000190
Bradi3g49260.1	bradi3g49260	phenylalanine ammonia-lyase, putative, expressed	PAL ORTHO000087 HOM000613
Bradi2g08350.1	bradi2g08350	receptor-like protein kinase 5 precursor, putative, expressed	Pkinase Pkinase_Tyr ORTHO000365 HOM000002
Bradi1g72762.1	bradi1g72762	hydrolase, alpha/beta fold family protein, putative, expressed	ORTHO021891 HOM000450
Bradi1g45710.1	bradi1g45710	plastocyanin-like domain containing protein, putative, expressed	Cu_bind_like ORTHO002673 HOM000055
Bradi5g10017.1	bradi5g10017	FAD binding domain containing protein, expressed	ORTHO006855 HOM000691
Bradi2g10820.1	bradi2g10820	BRASSINOSTEROID INSENSITIVE 1-associated receptor kinase 1 precursor, putative, expressed	LRRNT_2 LRR_1 ORTHO005827 HOM000004
Bradi1g54280.1	bradi1g54280	expressed protein	Pkinase Pkinase_Tyr ORTHO007931 HOM000002
Bradi3g03460.1	bradi3g03460	flavonol sulfotransferase, putative, expressed	Sulfotransfer_1 ORTHO026625 HOM000207
Bradi3g48530.1	bradi3g48530	transferase family protein, putative, expressed	Transferase ORTHO001336 HOM000062
Bradi4g21790.1	bradi4g21790	peptide transporter PTR2, putative, expressed	PTR2 ORTHO002126 HOM000031
Bradi1g13680.1	bradi1g13680	nuclear transcription factor Y subunit, putative, expressed	CBFB_NFYA ORTHO003967 HOM000553
Bradi1g26110.1	bradi1g26110	expressed protein	Tmemb_14 HOM001862
Bradi1g72350.1	bradi1g72350	glycosyl transferase, putative, expressed	Glyco_transf_8 ORTHO005972 HOM001012
Bradi2g34470.1	bradi2g34470	solute carrier family 35 member E3, putative, expressed	TPT UAA ORTHO001637 HOM000490
Bradi3g47950.1	bradi3g47950	expressed protein	Ank ORTHO001122 HOM002074
Bradi5g15490.1	bradi5g15490	ARPC2B, putative, expressed	ORTHO008608 HOM002240
Bradi5g20130.1	bradi5g20130	MYB family transcription factor, putative, expressed	ORTHO004899 HOM000007
Bradi4g04420.1	bradi4g04420	boron transporter protein, putative, expressed	HCO3_cotransp ORTHO000099 HOM000723
Bradi2g40437.1	bradi2g40437	GLUCAN SYNTHASE-LIKE protein, putative, expressed	ORTHO032426 HOM000178
Bradi2g22170.1	bradi2g22170	expressed protein	ORTHO009711 HOM004781
Bradi1g45487.1	bradi1g45487	annexin, putative, expressed	ORTHO011226 HOM000320
Bradi4g30540.1	bradi4g30540	CESA9 - cellulose synthase, expressed	Cellulose_synt Glycos_transf_2 ORTHO000003 HOM000082
Bradi1g50050.1	bradi1g50050	lung seven transmembrane domain containing protein, putative, expressed	Lung_7-TM_R ORTHO004651 HOM001242
Bradi2g08790.1	bradi2g08790	Cupin domain containing protein, expressed	Cupin_1 ORTHO000182 HOM000084
Bradi2g00220.1	bradi2g00220	fasciclin domain containing protein, expressed	Fasciclin ORTHO006591 HOM000419
Bradi2g37970.1	bradi2g37970	glycosyltransferase family 43 protein, putative, expressed	Glyco_transf_43 ORTHO003069 HOM001149
Bradi1g64650.1	bradi1g64650	expressed protein	Endosulfine HOM000751
Bradi3g54370.1	bradi3g54370	heparanase-like protein precursor, putative, expressed	Glyco_hydro_79n ORTHO001118 HOM001114

Bradi1g31320.1	bradi1g31320	AMP-binding domain containing protein, expressed	AMP-binding ORTHO000228 HOM000212
Bradi4g37490.1	bradi4g37490	OsFBK20 - F-box domain and kelch repeat containing protein, expressed	ORTHO006764 HOM005459
Bradi3g36217.1	bradi3g36217	endonuclease/exonuclease/phosphatase family domain containing protein, expressed	ORTHO019480 HOM000275
Bradi2g10970.1	bradi2g10970	tubulin/FtsZ domain containing protein, putative, expressed	Tubulin Tubulin_C ORTHO000021 HOM000128
Bradi2g55730.1	bradi2g55730	cytochrome P450, putative, expressed	AATase Condensation Transferase ORTHO007979 HOM003488
Bradi3g05750.1	bradi3g05750	AMP-binding domain containing protein, expressed	AMP-binding ORTHO000228 HOM000212
Bradi1g19660.1	bradi1g19660	uncharacterized GPI-anchored protein At5g19240 precursor, putative, expressed	ORTHO004354 HOM001766
Bradi2g27060.1	bradi2g27060	golgi transport complex protein-related, putative, expressed	COG5 ORTHO002975 HOM003421
Bradi4g40570.1	bradi4g40570	amino acid transporter, putative, expressed	ORTHO023276 HOM000159
Bradi1g68040.1	bradi1g68040	UDP-glucuronate 4-epimerase, putative, expressed	Epimerase ORTHO019135 HOM000461
Bradi1g06560.1	bradi1g06560	glycosyltransferase protein, putative, expressed	DUF563 ORTHO014729 HOM000406
Bradi1g32850.1	bradi1g32850	RIC10, putative, expressed	PBD HOM001247
Bradi1g32920.1	bradi1g32920	nodulin MtN3 family protein, putative, expressed	MtN3_slv ORTHO003565 HOM000160
Bradi1g14050.1	bradi1g14050	uncharacterized protein At4g06744 precursor, putative, expressed	LRRNT_2 ORTHO018969 HOM000308
Bradi3g56020.1	bradi3g56020	aquaporin protein, putative, expressed	MIP ORTHO012227 HOM000085
Bradi2g52470.1	bradi2g52470	ICE-like protease p20 domain containing protein, putative, expressed	Peptidase_C14 ORTHO001675 HOM001373
Bradi2g59400.1	bradi2g59400	exostosin family domain containing protein, expressed	Exostosin ORTHO000379 HOM001006
Bradi1g17830.1	bradi1g17830	potassium transporter, putative, expressed	K_trans ORTHO000022 HOM000120
Bradi2g48280.1	bradi2g48280	systemin receptor SR160 precursor, putative, expressed	Pkinase Pkinase_Tyr ORTHO000302 HOM000002
Bradi1g67870.1	bradi1g67870	expressed protein	ORTHO008245 HOM004952
Bradi3g32180.1	bradi3g32180	expressed protein	ORTHO004958 HOM003925
Bradi4g44860.1	bradi4g44860	PMR5, putative, expressed	ORTHO013293 HOM000050
Bradi1g67460.1	bradi1g67460	phospholipase A2, putative, expressed	Phospholip_A2_1 HOM000828
Bradi3g37530.1	bradi3g37530	ferric reductase, putative, expressed	FAD_binding_8 Ferric_reduct NADPH_Ox NAD_binding_6 ORTHO000035 HOM000327
Bradi1g06800.1	bradi1g06800	tubulin/FtsZ domain containing protein, putative, expressed	Misat_Myo_SegII Tubulin Tubulin_C ORTHO000021 HOM000128
Bradi2g36910.1	bradi2g36910	transferase family protein, putative, expressed	Transferase ORTHO022232 HOM000062
Bradi1g65750.1	bradi1g65750	glycosyltransferase family 43 protein, putative, expressed	Glyco_transf_43 ORTHO003069 HOM001149
Bradi1g65530.1	bradi1g65530	expressed protein	DUF231 ORTHO005860 HOM000050
Bradi1g75940.1	bradi1g75940	protein phosphatase 2C, putative, expressed	PP2C ORTHO013745 HOM000435
Bradi2g05927.1	bradi2g05927	harpin-induced protein 1 domain containing protein, expressed	ORTHO019195 HOM002559
Bradi2g49912.1	bradi2g49912	CESA4 - cellulose synthase, expressed	ORTHO000003 HOM000082
Bradi2g12150.2	bradi2g12150	S-adenosylmethionine synthetase, putative, expressed	S-AdoMet_synt_C S-AdoMet_synt_M S-AdoMet_synt_N ORTHO000163 HOM000932
Bradi3g58730.1	bradi3g58730	dehydration response related protein, putative, expressed	DUF248 ORTHO000772 HOM000110
Bradi2g59410.1	bradi2g59410	exostosin family domain containing protein, expressed	Exostosin ORTHO000379 HOM001006
