# Supplementary material for: Coordinated regulation of the entry and exit steps of aromatic amino acid biosynthesis supports the dual lignin pathway in grasses
Source: Nat Commun. 2023 Nov 9;14:7242. doi: 10.1038/s41467-023-42587-7 (PMC10636026; doi:10.1038/s41467-023-42587-7)
Supplement: Supplementary file 5 — Supplementary Data 2 [file 41467_2023_42587_MOESM5_ESM.pdf]

Supplemental Table S2. List of primers used in this study.

Primer name	sequence (from 5'-)	Target gene	Purpose
pHM532Bd347pML94F	GATCTAGACTCGAGGGTACCATGCTGTCGTCTTCC	*BdTyrA1*	In-Fusion cloning into pML94 for transient expression in Arabidopsis protoplasts
pHM533Bd347pML94R	CTAGTGCATGCGGCCGCATTCCGGACGGTGG	*BdTyrA1*	In-Fusion cloning into pML94 for transient expression in Arabidopsis protoplasts
pHM534Bd347pET28aF	CGCGCGGCAGCCATATGCGCGCCATCGACGC	*BdTyrA1*	In-Fusion cloning into NdeI/BamHI sites of pET28a
pHM535Bd347pET28aR	GCTCGAATTCGGATCCCTAATTCCGGACGGTGG	*BdTyrA1*	In-Fusion cloning into NdeI/BamHI sites of pET28a
pHM536Bd348pML94F	GATCTAGACTCGAGGGTACCATGTCTTCCTCCGGTC	*BdTyrA2*	In-Fusion cloning into pML94 for transient expression in Arabidopsis protoplasts
pHM537Bd348pML94R	CTAGTGCATGCGGCCGCCCGTCCGTCATCCAA	*BdTyrA2*	In-Fusion cloning into pML94 for transient expression in Arabidopsis protoplasts
pHM538Bd348pET28aF	CGCGCGGCAGCCATATGCGTGCCACGGACGC	*BdTyrA2*	In-Fusion cloning into NdeI/BamHI sites of pET28a
pHM539Bd348pET28aR	GCTCGAATTCGGATCCTCACCGTCCGTCATCC	*BdTyrA2*	In-Fusion cloning into NdeI/BamHI sites of pET28a
pHM540Bd39pML94F	GATCTAGACTCGAGGGTACCATGGCTTCCTCCCTTG	*BdTyrAnc*	infusion cloning into pML94 for transient expression in Arabidopsis protoplasts
pHM541Bd39pML94R	CTAGTGCATGCGGCCGCAATGGGGACCCTCTCT	*BdTyrAnc*	infusion cloning into pML94 for transient expression in Arabidopsis protoplasts
pHM542Bd39pET28aF	CGCGCGGCAGCCATATGGCCGAGCAGGAGCAA	*BdTyrAnc*	In-Fusion cloning into NdeI/BamHI sites of pET28a
pHM543Bd39pET28aR	GCTCGAATTCGGATCCTTAAATGGGGACCCTCTC	*BdTyrAnc*	In-Fusion cloning into NdeI/BamHI sites of pET28a
pHM1751-SbTyrA1GGF	TCACTCTGTGGTCTCAAATGCGCGCGCTGGACGCCGCCC	*SbTyrA1*	In-Fusion cloning into pAGM1287 BsaI sites as Golden Gate level 0 part
pHM1752-SbTyrA1GGR	CCACTTCGTGGTCTCACGAACTCTTGCGGACGTTGGAGGAG	*SbTyrA1*	In-Fusion cloning into pAGM1287 BsaI sites as Golden Gate level 0 part
pHM1753-SbTyrA2GGF	TCACTCTGTGGTCTCAAATGCGCGCCACGGGTGCCTCGC	*SbTyrA2*	In-Fusion cloning into pAGM1287 BsaI sites as Golden Gate level 0 part
pHM1754-SbTyrA2GGR	CCACTTCGTGGTCTCACGAACTATTATTCTCCTCTCCCGACTTG	*SbTyrA2*	In-Fusion cloning into pAGM1287 BsaI sites as Golden Gate level 0 part
pHM1755-SbTyrA3GGF	TCACTCTGTGGTCTCAAATGAGCCCGCCCGCCGCCACCGC	*SbTyrAnc*	In-Fusion cloning into pAGM1287 BsaI sites as Golden Gate level 0 part
pHM1756-SbTyrA3GGR	CCACTTCGTGGTCTCACGAACTTAAGAGGCGAGCTGCAGGAG	*SbTyrAnc*	In-Fusion cloning into pAGM1287 BsaI sites as Golden Gate level 0 part
pHM1757-SbTyrA3nestF	ATGGCCTCCTCGCTCCGCC	*SbTyrAnc*	Nested PCR for Sorghum TyrAnc
pHM1758-SbTyrA3nestR	GTATCCGGTTGAAGTGTAGG	*SbTyrAnc*	Nested PCR for Sorghum TyrAnc
pHM2274_BdTyrA1_qF	CACCACCGTCCGGAATTAGC	*BdTyrA1*	RT-qPCR
pHM2275_BdTyrA1_qR	GCACCAGTTTCTCCCCAAAG	*BdTyrA1*	RT-qPCR
pHM2316 BdTyrA2 qF2	GATGACGGACGGTGATCTCG	*BdTyrA2*	RT-qPCR
pHM2317 BdTyrA2 qR2	TTCGTACCGCTTGTTGGTCG	*BdTyrA2*	RT-qPCR
pHM2278_BdTyrA3_qF	TGCTGTGTCCCCTCTCCTC	*BdTyrAnc*	RT-qPCR
pHM2279_BdTyrA3_qR	AGGGCTGAAAGACACTGGGC	*BdTyrAnc*	RT-qPCR
pHM2318 SvTyrA2 qF2	CAGACAATGCGGAGATGATCG	*SvTyrA2* (Sevir.4G287000.1)	RT-qPCR
pHM2319 SvTyrA2 qR2	TTTGCTTCAGAAACCATGTCAC	*SvTyrA2* (Sevir.4G287000.1)	RT-qPCR
pHM2282_SvTyrA1_qF	GGTAGTAATTCAGTGCGTCGG	*SvTyrA1* (Sevir.4G286800.1)	RT-qPCR
pHM2283_SvTyrA1_qR	GGTGTTCTTCTTCCAGAGAGG	*SvTyrA1* (Sevir.4G286800.1)	RT-qPCR
pHM2284_SvTyrA3_qF	GATCGCTTCCATCCCAAGGC	*SvTyrAnc* (Sevir.4G143200.1)	RT-qPCR

pHM2285_SvTyrA3_qR	CAGGCGGTCTGAAAGGAAGG	*SvTyrAnc* (Sevir.4G143200.1)	RT-qPCR
pHM2290_BdUBI10_qF	AGTTGTCGCGTGTCTGAGTC	Bradi1g32860.3	RT-qPCR, reference gene Polyubiquitin 10
pHM2291_BdUBI10_qR	ACACGGGCTCACTTATTCATC	Bradi1g32860.3	RT-qPCR, reference gene Polyubiquitin 10
pHM2294_SvUBI4_qF	GGGCTCATTGTGCTGCTGTC	Sevir.5G079801.1	RT-qPCR, reference gene Polyubiquitin 4
pHM2295_SvUBI4_qR	CCGGAGGACATAGGACTTGC	Sevir.5G079801.1	RT-qPCR, reference gene Polyubiquitin 4
pHM2178 BdDHS1b Fwd	GGTGCCGCGCGGCAGCCATATGGCCGTCCACGCCGCGGAGCC	*BdDHS1b*	In-Fusion cloning into NdeI/BamHI sites of pET28a
pHM2179 BdDHS1b Rvs	CGGAGCTCGAATTCGGATCCTCAGAAACCATAGGTTGGCAATG	*BdDHS1b*	In-Fusion cloning into NdeI/BamHI sites of pET28a
pHM2180 BdDHS1a Fwd	GGTGCCGCGCGGCAGCCATATGGCCGTGCACGCCGCCGACCC	*BdDHS1a*	In-Fusion cloning into NdeI/BamHI sites of pET28a
pHM2181 BdDHS1a Rvs	CGGAGCTCGAATTCGGATCCTTAGAAGGCCAATGGCGGCAGTG	*BdDHS1a*	In-Fusion cloning into NdeI/BamHI sites of pET28a
pHM2184 BdDHS2 Fwd	*GGTGCCGCGCGGCAGCCATATGATCCGCGCGCACGCGGTGCG*	*BdDHS2*	In-Fusion cloning into NdeI/BamHI sites of pET28a
pHM2185 BdDHS2 Rvs	*CGGAGCTCGAATTCGGATCCTCAGAGTCCCATGGATGATGG*	*BdDHS2*	In-Fusion cloning into NdeI/BamHI sites of pET28a
pHM2182 BdDHSnc Fwd	*GGTGCCGCGCGGCAGCCATATGCGCGCGACGTCGGTCGCGGC*	*BdDHSnc*	In-Fusion cloning into NdeI/BamHI sites of pET28a
pHM2183 BdDHSnc Rvs	CGGAGCTCGAATTCGGATCCTTAAGCTTCTACTCTAGATATCAAGC	*BdDHSnc*	In-Fusion cloning into NdeI/BamHI sites of pET28a
pHM2342 SbDHS1a Fwd	GGTGCCGCGCGGCAGCCATATGGCCATCCACGCCGCCGACCC	*SbDHS1a*	In-Fusion cloning into NdeI/BamHI sites of pET28a
pHM2343 SbDHS1a Rvs	CGGAGCTCGAATTCGGATCCTCAGAAAGCCAGTGGTGGCAGC	*SbDHS1a*	In-Fusion cloning into NdeI/BamHI sites of pET28a
pHM2344 SbDHS2 Fwd	GGTGCCGCGCGGCAGCCATATGCTCCGCGCCCGCGCCGTCC	*SbDHS2*	In-Fusion cloning into NdeI/BamHI sites of pET28a
pHM2345 SbDHS2 Rvs	CGGAGCTCGAATTCGGATCCTCACAGACGAATGGAACCCAGC	*SbDHS2*	In-Fusion cloning into NdeI/BamHI sites of pET28a
pHM2675 BdDHS3a GGF	TCACTCTGTGGTCTCAAATGGCCGTGCACGCCGCCG	*BdDHS1a*	In-Fusion cloning into pAGM1287 BsaI sites as Golden Gate level 0 part
pHM2676 BdDHS3a GGR	CCACTTCGTGGTCTCACGAACTGAAGGCCAATGGCGGC	*BdDHS1a*	In-Fusion cloning into pAGM1287 BsaI sites as Golden Gate level 0 part
pHM2546 BdDHS3b GGF	TCACTCTGTGGTCTCAAATGGCCGTCCACGCCGCGGAGCC	*BdDHS1b*	In-Fusion cloning into pAGM1287 BsaI sites as Golden Gate level 0 part
pHM2547 BdDHS3b GGF	CCACTTCGTGGTCTCACGAACTGAAACCATAGGTTGGCAATG	*BdDHS1b*	In-Fusion cloning into pAGM1287 BsaI sites as Golden Gate level 0 part
pHM2677 BdDHS3c GGF	TCACTCTGTGGTCTCAAATGATCCGCGCGCACGCGG	*BdDHS2*	In-Fusion cloning into pAGM1287 BsaI sites as Golden Gate level 0 part
pHM2678 BdDHS3c GGR	CCACTTCGTGGTCTCACGAACTGAGTCCCATGGATGATG	*BdDHS2*	In-Fusion cloning into pAGM1287 BsaI sites as Golden Gate level 0 part
