# Supplementary material for: Coordinated regulation of the entry and exit steps of aromatic amino acid biosynthesis supports the dual lignin pathway in grasses
Source: Nat Commun. 2023 Nov 9;14:7242. doi: 10.1038/s41467-023-42587-7 (PMC10636026; doi:10.1038/s41467-023-42587-7)
Supplement: Supplementary file 6 — Reporting Summary [file 41467_2023_42587_MOESM6_ESM.pdf]

nature portfolio

Corresponding author(s): Hiroshi A. Maeda

Last updated by author(s): Sep 28, 2023

Reporting Summary

Nature Portfolio wishes to improve the reproducibility of the work that we publish. This form provides structure for consistency and transparency in reporting. For further information on Nature Portfolio policies, see our Editorial Policies and the Editorial Policy Checklist.

Statistics

For all statistical analyses, confirm that the following items are present in the figure legend, table legend, main text, or Methods section.

n/a	Confirmed	
☐	☒	The exact sample size (*n*) for each experimental group/condition, given as a discrete number and unit of measurement
☐	☒	A statement on whether measurements were taken from distinct samples or whether the same sample was measured repeatedly
☐	☒	The statistical test(s) used AND whether they are one- or two-sided<br>*Only common tests should be described solely by name; describe more complex techniques in the Methods section.*
☒	☐	A description of all covariates tested
☒	☐	A description of any assumptions or corrections, such as tests of normality and adjustment for multiple comparisons
☐	☒	A full description of the statistical parameters including central tendency (e.g. means) or other basic estimates (e.g. regression coefficient) AND variation (e.g. standard deviation) or associated estimates of uncertainty (e.g. confidence intervals)
☐	☒	For null hypothesis testing, the test statistic (e.g. *F*, *t*, *r*) with confidence intervals, effect sizes, degrees of freedom and *P* value noted<br>*Give P values as exact values whenever suitable.*
☒	☐	For Bayesian analysis, information on the choice of priors and Markov chain Monte Carlo settings
☒	☐	For hierarchical and complex designs, identification of the appropriate level for tests and full reporting of outcomes
☒	☐	Estimates of effect sizes (e.g. Cohen's *d*, Pearson's *r*), indicating how they were calculated

Our web collection on statistics for biologists contains articles on many of the points above.

Software and code

Policy information about availability of computer code

Data collection	Microsoft 365 Excel was used for data collection for this study.
Data analysis	Statistical analysis integrated in the Microsoft 365 Excel was used for data analysis in this study. Phylogenetic trees were reconstructed using MEGA-11. Image-based protein quantification by immunoblot was performed using ImageJ v1.52a in combination with Microsoft 365 Excel.

For manuscripts utilizing custom algorithms or software that are central to the research but not yet described in published literature, software must be made available to editors and reviewers. We strongly encourage code deposition in a community repository (e.g. GitHub). See the Nature Portfolio guidelines for submitting code & software for further information.

Data

Policy information about availability of data

All manuscripts must include a data availability statement. This statement should provide the following information, where applicable:

- Accession codes, unique identifiers, or web links for publicly available datasets
- A description of any restrictions on data availability
- For clinical datasets or third party data, please ensure that the statement adheres to our policy

The original data supporting the findings of this study are provided with this paper as a Source Data File. All published data are available without restrictions. The sequence of the genes studied in this work are publicly available at Phytozome v13, and their IDs clearly indicated at the first time introduced.

Human research participants

Policy information about studies involving human research participants and Sex and Gender in Research.

Reporting on sex and gender	N/A
Population characteristics	N/A
Recruitment	N/A
Ethics oversight	N/A

Note that full information on the approval of the study protocol must also be provided in the manuscript.

Field-specific reporting

Please select the one below that is the best fit for your research. If you are not sure, read the appropriate sections before making your selection.

☒ Life sciences ☐ Behavioural & social sciences ☐ Ecological, evolutionary & environmental sciences

For a reference copy of the document with all sections, see nature.com/documents/nr-reporting-summary-flat.pdf

Life sciences study design

All studies must disclose on these points even when the disclosure is negative.

Sample size	In planta metabolite analyses were conducted with 5 to 6 independent biological replications to account for biological variations of the system. In vitro enzyme characterization was conducted with at least two independent experiments, using different batches of purified enzyme. The time course 13C labeling experiments were conducted in duplicates considering the cost and limited space available in the labeling chamber and also different time points for each genotype serve as additional independent measurements to evaluate the overall rate of 13C incorporation.
Data exclusions	No data was excluded from the analyses, except for figures 3g and 5b-c, where one datapoint was excluded due to technical issues while processing or analyzing the samples. The exclusion of these datapoints has been clearly indicated in the figure legends. Original data corresponding to these excluded datapoints are provided within the Source Data File.
Replication	Each experiments were replicated at least twice or more and overall consistent results were obtained.
Randomization	Plant samples used in the study were randomized to avoid interference of environmental variations.
Blinding	The investigator were not blinded to sample allocations but used the above randomization and positive/negative controls to carefully evaluate genotypic variations (or between different enzymes).

Reporting for specific materials, systems and methods

We require information from authors about some types of materials, experimental systems and methods used in many studies. Here, indicate whether each material, system or method listed is relevant to your study. If you are not sure if a list item applies to your research, read the appropriate section before selecting a response.

Materials & experimental systems

n/a	Involved in the study	
☒	☐	Antibodies
☒	☐	Eukaryotic cell lines
☒	☐	Palaeontology and archaeology
☐	☒	Animals and other organisms
☒	☐	Clinical data
☒	☐	Dual use research of concern

Methods

n/a	Involved in the study	
☒	☐	ChIP-seq
☒	☐	Flow cytometry
☒	☐	MRI-based neuroimaging

Animals and other research organisms

Policy information about studies involving animals; ARRIVE guidelines recommended for reporting animal research, and Sex and Gender in Research

Laboratory animals	N/A
Wild animals	N/A
Reporting on sex	N/A
Field-collected samples	N/A
Ethics oversight	No animals were used in this study and no oversight was required in this project.

Note that full information on the approval of the study protocol must also be provided in the manuscript.
